# Editorial: CRISPR-based biosensors

**DOI:** 10.3389/fbioe.2022.1060249

**Published:** 2022-10-28

**Authors:** Long Ma, Yaru Li

**Affiliations:** State Key Laboratory of Food Nutrition and Safety, Key Laboratory of Industrial Microbiology, Ministry of Education, Tianjin Key Laboratory of Industry Microbiology, National and Local United Engineering Lab of Metabolic Control Fermentation Technology, China International Science and Technology Cooperation Base of Food Nutrition/Safety and Medicinal Chemistry, College of Biotechnology, Tianjin University of Science and Technology, Tianjin, China

**Keywords:** CRISPR-Cas, biosensors, point-of-care testing (POCT), CRISPR-Dx, trans-cleavage

CRISPR/Cas was firstly discovered in the 1980s and was recognized as the adaptive immune system, which is evolved to fight against invading nucleic acids to obtain self-immunity for prokaryotic cells. The discovery of CRISPR mediated adaptive immunity and a range of CRISPR-associated proteins (Cas) have led to transformative advances in genome editing and revolutionized the field of genome engineering, which was recognized by the Nobel Prize in Chemistry in 2020. Moreover, CRISPR/Cas demonstrated its significant potential in next-generation biosensing owing to the unique properties such as programmability, high specificity, high sensitivity, single-base resolution, and target-induced trans-cleavage. The milestone work of CRISPR/Cas biosensing, namely SHERLOCK was reported in the year of 2017. A rapid development of CRISPR/Cas biosensing has be witnessed (Li et al., 2019; Aman et al.; Chen et al.; Li et al., 2022; Shi et al.; Yin et al., 2022; Zhang et al.). Then a new term CRISPR-Dx (CRISPR-based diagnostics) has been coined, which is developed rapidly to detect a wide range of targets spanning nucleic acids to non-nucleic acids targets including ions, enzymes, small molecules, transcription factors, cells and etc. With the coming-of-age of CRISPR-Dx, it is time to recollect, rethink and reflect on how this technology can be elaborated in order to overcome ongoing and futuristic challenges.

To further unlock the potential of the CRISPR/Cas, researchers have developed a range of platforms integrating CRISPR/Cas with various technologies. Such integrated solutions could enable rapid, specific, low-cost, easy-to-deploy, on-site, automatic, miniatured, multi-targeted, custom-tailored detection of analytes in a smart, interoperable, and portable manner.

This Research Topic gathered innovative approaches of CRISPR/Cas (like Cas12a, Cas12b) based biosensors *via* diverse readouts, such as fluorescent signal upon lateral flow strip, LED blue light. Certain microbes and nematodes that are seriously harmful to human body or environment are taken as detection targets, including *S. flexneri* (Shi et al.), *A. besseyi* (Zhang et al.), SARS-CoV-2 (Aman et al.). All of them were presented with potential at the POCT. In particular:

Shi et al. integrated CRISPR/Cas12a with loop-mediated isothermal amplification for the diagnosis of *S. flexneri* by naked eyes with a total time of 40 min. The platform was proven to be technically sound for the reliable and quick diagnosis of *S. flexneri*.

Zhang et al. implemented lateral flow strip assay to make the detection result visualize by naked eyes and improve the user-friendliness. Recombinase polymerase amplification was introduced as well to detect *A. besseyi* with a limit of detection of 1,000 copies/μL plasmid in 45 min, thereby enabling a rapid, sensitive, and specific detection system without sophisticated equipment for on-site surveillance of *A. besseyi*.

Aman et al. developed and optimized iSCAN-V2, a one-pot reverse transcription-recombinase polymerase amplification-coupled CRISPR/Cas12b-based assay for SARS-CoV-2 detection in less than an hour. Besides, the platform was coupled with a low-cost, commercially available fluorescence visualizer to enable its in-field deployment, offering an alternative to performing a POCT in low-resource settings.

Chen et al. reviewed recent advances from nucleic acids to other non-nucleic small molecules or analytes. The authors provided a synopsis of CRISPR biosensing strategies and presented the challenges and perspectives of CRISPR biosensors.

Currently, only a few CRISPR/Cas biosensing for multiplexed detection have been reported. The amplification process was necessary for the large portion of CRISPR/Cas biosensing, and there is the influence of sample pre-treatments and sample matrix. We hope this Research Topic contributes valuable insights into CRISPR-Cas based biosensors as an emerging toolbox with simplicity, rapidity, adaptability, sensitivity, specificity and on-site capability. This will assist and inspire researchers to conduct futuristic research beyond the state-of-the-art to further propel this technology.
